# Acute Effects of Different Training Sessions on the Extended Blood Steroid Profile in Elite Male Football Players

**DOI:** 10.1007/s40279-026-02467-8

**Published:** 2026-06-02

**Authors:** Federico Ponzetto, Alberto Franceschi, Laura Leoni, Alessia Melis, Giulia Montesano, Andrea Marchini, Fabio Settanni, Raul Nicoli, Gianluca Aimaretti, Andrea Benso, Tiia Kuuranne, Mirko Parasiliti-Caprino

**Affiliations:** 1 Clinical Biochemistry Laboratory, Department of Medical Sciences, University of Turin, Turin, Italy; 2 Sport Science and R&D Department, Juventus Football Club, Turin, Italy; 3 Research Institute for Sport and Exercise Sciences, Liverpool John Moores University, Liverpool, UK; 4 Endocrinology, Diabetes and Metabolism, Department of Medical Sciences, University of Turin, City of Health and Science University Hospital, Turin, Italy; 5 J Medical, Turin, Italy; 6 Clinical Biochemistry Laboratory, City of Health and Science University Hospital, Turin, Italy; 7 Swiss Laboratory for Doping Analyses, University Center of Legal Medicine Geneva and Lausanne, Centre Hospitalier Universitaire Vaudois and University of Lausanne, Lausanne, Switzerland

## Abstract

**Background:**

Acute exercise is a recognized physiological confounder of circulating steroid hormones, yet evidence on how real-world football training affects the blood steroid profile (BSP) within the Athlete Biological Passport (ABP) remains limited in elite athletes.

**Objective:**

To examine acute endocrine response to football training sessions of differing load and to explore their association with perceived exertion.

**Methods:**

A total of 27 elite male professional outfield football players from an Italian Serie A club were assessed during the in-season period (2022–2023 competitive season). Each player completed one predefined low-load (LL), moderate-load (ML), or high-load (HL) training session. Venous blood samples were collected immediately before and after training. Collectively, 26 circulating steroids were quantified by liquid chromatography–tandem mass spectrometry. These included both unconjugated hormones and phase II metabolites relevant to the BSP. External training load was assessed using global navigation satellite systems, while internal load was quantified via heart-rate telemetry and rating of perceived exertion (RPE). Data were analyzed using nonparametric statistics, linear mixed-effects models, and multivariable logistic regression.

**Results:**

Training elicited significant, load-associated alterations in circulating BSP steroids. HL sessions induced marked post-training increases in free glucocorticoids including cortisone (median Δ + 7.4 ng/mL), and unconjugated androgens such as androstenedione (median Δ + 131.3 pg/mL), whereas LL sessions induced minimal changes. The testosterone-to-androstenedione ratio decreased following ML and HL training (median 8.2–6.6, *p* < 0.001). Athletes reporting higher perceived exertion (RPE ≥ 4) exhibited greater endocrine perturbations, with significantly larger increases in cortisol-related steroids (e.g., 11-deoxycortisol, *p* < 0.001).

**Conclusions:**

Acute football training is associated with rapid modulation of circulating steroid hormones, particularly following higher training loads and perceived exertion. These findings highlight the potential relevance of recent training exposure when interpreting BSP variations and suggest that integrating steroidomic profiles with training-load information may improve the physiological contextualization of antidoping biomarkers.

**Supplementary Information:**

The online version contains supplementary material available at https://doi.org/10.1007/s40279-026-02467-8.

## Key Points

**Table:** 

This study shows that football training sessions of different volume and intensity elicit distinct acute hormonal responses in elite male players.
High-load sessions induced marked changes in several steroid hormones, whereas low-load sessions had minimal effects.
Combining training-load metrics with advanced steroidomic profiling may provide valuable insight into athletes’ physiological stress and recovery.

## Introduction

Steroid hormones, including circulating androgens and corticosteroids in both unconjugated and conjugated forms, play a central role in the regulation of energy metabolism, immune function, and the physiological response to physical stress [1]. While the chronic effects of exercise training on endocrine function are well described, substantially less is known about the acute steroidomic responses elicited by structured training sessions performed in real-world elite sport settings [2, 3].

This gap is particularly relevant in football, a sport characterized by substantial variability in training load and frequent exposure to high-intensity exercise. Acute exercise activates the hypothalamic–pituitary–adrenal (HPA) axis and modulates androgen production and metabolism [4–7]; however, the magnitude and direction of these responses depend on training intensity, internal load, and perceived exertion [4, 8]. A detailed understanding of acute endocrine response may therefore provide valuable insight into physiological stress and short-term training adaptation in elite players.

In football-specific contexts, several studies have investigated endocrine responses to training and match load, primarily focusing on a limited set of hormonal markers such as testosterone, cortisol, and the testosterone-to-cortisol ratio [9–11]. These markers are widely used in applied settings as indicators of psychophysiological stress and recovery status. However, their responses are often variable and influenced by multiple factors, including training load, match density, individual fitness level, and non-sport-related stressors [12–14]. Moreover, most available evidence is based on salivary or single-hormone assessments, which may not fully capture the complexity of steroid regulation in response to exercise [15]. Recent reviews have therefore emphasized the need for more comprehensive and integrative approaches to endocrine monitoring in football, capable of accounting for the multidimensional nature of hormonal responses and their high interindividual variability [14].

Beyond performance and recovery considerations, acute exercise-induced endocrine fluctuations are also highly relevant for antidoping applications. The misuse of endogenous anabolic androgenic steroids has traditionally been monitored using urinary steroid profiling, from the testosterone/epitestosterone ratio [16, 17] to the steroidal module of the Athlete Biological Passport (ABP) [18, 19]. More recently, blood-based steroid markers have been introduced through the blood steroid profile (BSP), expanding the ABP framework and improving sensitivity for specific doping practices [20, 21]. Compared with urinary profiling, however, the physiological confounders influencing the BSP, particularly acute exercise, remain insufficiently characterized [22].

Advances in mass spectrometry now enable comprehensive blood steroid profiling, including both unconjugated hormones and phase II metabolites [23]. In addition to their relevance as antidoping biomarkers [24], phase II metabolites may reflect acute modulation of steroid metabolism and clearance pathways in response to exercise-induced stress [25]. Despite this potential, data describing the acute response of both unconjugated and conjugated steroids to structured football training are scarce, especially in elite athletes routinely subjected to post-exercise blood sampling [2, 3, 26, 27].

On the basis of the existing evidence, we hypothesized that higher training loads and greater perceived exertion would be associated with larger perturbations in circulating steroid hormones, particularly in glucocorticoid and androgen pathways, and with measurable alterations in key BSP-related markers such as the testosterone-to-androstenedione ratio. The present study therefore aimed to examine the acute endocrine response to football training sessions of differing load and intensity using a comprehensive panel of circulating steroids in elite male professional football players, and to clarify how training load and perceived exertion influence key components of the BSP relevant to the ABP.

## Subjects and Methods

### Study Design and Participants

This observational study included 27 outfield male professional football players (age: 19 ± 2 years; body mass: 78 ± 7 kg; height: 184 ± 6 cm) from the under-19 and under-23 squads of an Italian Serie A club. Players were representative of all positional roles: central defenders (*n* = 6), wide defenders (*n* = 6), central midfielders (*n* = 4), wide midfielders (*n* = 6), and attackers (*n* = 5) and were classified as elite level (tier 4) according to the performance classification framework proposed by McKay et al. [28]. Participants were eligible if free from medical conditions and medications known to affect endocrine function. Blood samples were collected immediately before and after a single training session, with a 3-h maximum interval between assessments, during the in-season period. Each player completed one predefined training session classified as low-load (LL), moderate-load (ML), or high-load (HL). Anthropometric data and training-load measures were collected on the day of the session using global navigation satellite systems (GNSS), heart-rate monitoring, and rating of perceived exertion (RPE). Participants were familiar with the training and monitoring practice and were instructed to maintain their normal dietary intake during the study. The study was conducted in accordance with the Strengthening the Reporting of Observational studies in Epidemiology (STROBE) guidelines [29] and approved by the Ethics Committee of the University of Turin (approval no. 186676).

### Training-Session Characteristics and Quantification of Training Load

Players completed one predefined training session classified as LL, ML, or HL, according to planned duration, structure, and expected physiological demand. The three sessions were scheduled and performed during the central days of a competitive microcycle to reduce the influence of contextual match factors such as participation status (e.g., starters, nonstarters) and related fatigue (e.g., prolonged recovery of performance capacity and physiological state in the days after a match). LL sessions were characterized by reduced volume and intensity, moderate-load sessions by intermediate demands, and HL sessions by longer duration and a greater proportion of high-intensity activities. Detailed information on training session content and structure is reported in the Supplementary Material (Sect. 1.1).

External training load was quantified using global navigation satellite systems (GNSS; Apex Pro Series, 10 Hz, STATSports, Newry, Northern Ireland), while internal load was assessed using heart-rate telemetry (Polar H10, Polar, Kempele, Finland) and session rating of perceived exertion (RPE) collected using Borg’s category ratio-10 scale [30]. GNSS- and heart-rate-derived data were processed using the manufacturer’s software, as previously described [31]. For the present research, external load variables included total distance, high-speed running distance, maximal speed, speed intensity, dynamic stress load, high metabolic load distance, and counts of high-intensity accelerations and decelerations. Internal load variables included average and maximal heart rate, time spent above 85% of maximal heart rate, heart-rate exertion, and RPE. These variables were selected on the basis of a recent conceptual framework for training load monitoring in football [32].

### Sample Collection and Steroid Analysis

Blood samples were collected on the same day immediately before and after the training session. Pre-training samples were collected between 2:30 p.m. and 3:00 p.m., whereas post-training samples were obtained as soon as practically feasible after session completion (approximately between 5:00 p.m. and 5:30 p.m.), with players sampled in a consistent order across sessions. Although minor variations in sampling time may have occurred owing to logistical constraints, the overall timing was comparable across participants. Blood-sampling procedures were designed to capture the acute endocrine response to training. Samples were collected immediately before and after training, with participants seated for at least 10 min prior to each blood draw. Venous blood was collected into SST™ II Advance BD Vacutainer tubes (6 mL; Becton, Dickinson & Company, Franklin Lakes, USA), following standard clinical procedures. The timing of sampling was intentionally selected during the study design to coincide with the mid-afternoon period, corresponding to the descending phase of adrenocorticotropic hormone (ACTH)-related steroid secretion, to reduce the potential confounding influence of circadian peaks. Venous blood was drawn into serum separator tubes, centrifuged after clotting, and serum aliquots were stored at − 20 °C for a maximum of 3 months prior to analysis. Particular care was taken to avoid repeated freeze–thaw cycles, which are known to potentially affect steroid concentrations.

A targeted ultra-high-performance liquid chromatography–tandem mass spectrometry (UHPLC–MS/MS) method was used to quantify circulating steroids, as previously described and validated for antidoping applications [33]. The analytical panel included 26 steroids encompassing unconjugated glucocorticoids and androgens as well as phase II androgen metabolites. The complete list of analytes is presented in Supplementary Table S1. Detailed information on sample preparation, chromatographic conditions, mass spectrometric parameters, and analytical performance is provided in the Supplementary Material (Sect. 1.2). To ensure analytical reliability, quality control samples at three concentration levels were included in each analytical batch, and all analyses were performed using standardized laboratory procedures.

### Statistical Analysis

Each player completed only one predefined training session. Sample size estimation was not appropriate for this work. This study was conducted within the set-up of a professional football club, providing the advantage of being able to evaluate the whole group of players per age group. We have, therefore, assessed the “entire population” of interest [34]. Although some variables did not significantly deviate from normality on the basis of formal tests (e.g. Shapiro–Wilk), a consistent nonparametric approach was adopted across all analyses. This choice was driven by the relatively small and unbalanced subgroup sizes, the limited power of normality tests in small samples, and the need to ensure methodological consistency across the large number of outcomes assessed. Nonparametric methods were therefore preferred as a more robust and conservative approach that does not rely on distributional assumptions. Continuous variables are reported as medians and interquartile ranges (IQR), while categorical variables are expressed as absolute counts and percentages.

For all analyses, changes (Δ) in serum steroid concentrations were calculated as post-training minus pretraining values; therefore, positive Δ values indicate an increase after training, whereas negative values indicate a decrease. Pre- and post-training comparisons of serum steroid concentrations were conducted using the Wilcoxon signed-rank test for paired data. Differences between independent groups, such as training types (LL, ML, and HL) and rate of perceived exertion (RPE < 4 versus RPE ≥ 4) categories, were assessed using the Kruskal–Wallis test for more than two groups or the Mann–Whitney *U* test for two-group comparisons. Categorical variables were analyzed using the chi-squared test or Fisher’s exact test, as appropriate.

To identify determinants of hormonal responses, we fitted linear mixed-effects models (LMM) for each steroid, with time (pre versus post), training (LL, ML, and HL), and time × training interaction as fixed effects and athlete ID as a random intercept. Relevant training-related covariates, including heart rate exertion, dynamic stress load (DSL), speed intensity, and RPE, were included as fixed covariates on the basis of physiological plausibility and univariate associations (*p* < 0.15). For steroids with poor LMM fit or convergence issues, standard linear regression models were applied using the same predictors and covariates. Detailed results of these complementary linear regression analyses are presented in Supplementary Table S2. Given the large number of analytes and comparisons, results should be interpreted with caution and considered exploratory.

In addition, a logistic regression model was constructed to identify steroid-based factors associated with RPE ≥ 4. Each model included steroid delta values and was adjusted for training type and telemetry covariates. Results were reported as odds ratios (OR) with 95% confidence intervals (CI). In multivariable models, assumptions were evaluated through visual inspection of residuals, and no major violations were observed.

All statistical analyses were performed using Stata version 18 (StataCorp, College Station, TX, USA). A two-tailed *p*-value < 0.05 was considered statistically significant.

## Results

### Study Population and Training-Load Characteristics

A total of 27 elite male football players (median age 19 years, IQR 2) were included in the analysis. Players completed one of three different training session types: LL (*n* = 6), ML (*n* = 11), or HL (*n* = 10). Both external and internal training load parameters differed across training modalities (Table 1).

**Table 1:** Training load parameters according to training modality

Variables/parameters	Total (*n* = 27)	High-load (*n* = 10)	Moderate-load (*n* = 11)	Low-load (*n* = 6)	*p*-value
Age (years)	19.0 (2.0)	19.5 (1.0)	18.0 (1.0)	20.0 (1.0)	**0.011**
Weight (kg)	76.0 (8.0)	76.0 (7.5)	74.4 (5.4)	80.8 (6.5)	0.159
BMI (kg/m^2^)	22.8 (2.1)	23.7 (1.6)	22.0 (2.0)	22.8 (1.6)	**0.035**
Total distance (m)	6098.7 (1509.4)	6529.5 (931.7)	6177.2 (783.2)	4320.9 (958.5)	**< 0.001**
Distance > 20 km/h (m)	150.1 (160.8)	190.9 (137.7)	177.4 (180.3)	73.7 (97.7)	0.065
Distance > 25 km/h (m)	9.8 (20.2)	7.2 (15.4)	26.8 (49.2)	1.4 (5.7)	**0.021**
Max speed (km/h)	26.8 (3.6)	26.9 (1.8)	29.0 (3.9)	24.3 (3.9)	**0.016**
Speed intensity (au)	292.2 (75.3)	313.9 (50.9)	298.4 (46.0)	199.9 (48.6)	**< 0.001**
Max heart rate (bpm)	183.0 (20.4)	190.0 (12.0)	179.0 (22.0)	172.3 (11.0)	**0.016**
Average heart rate (bpm)	137.3 (26.1)	142.6 (19.1)	137.3 (22.1)	122.9 (13.6)	0.089
Time > 85% HR max (%)	6.5 (24.6)	16.4 (24.9)	6.5 (24.4)	1.4 (3.1)	0.131
Heart rate exertion (au)	135.8 (121.7)	195.8 (125.7)	138.3 (87.7)	83.6 (13.6)	**0.016**
Dynamic stress load (au)	212.4 (193.9)	302.6 (140.6)	210.6 (149.6)	105.4 (32.6)	**0.003**
Accelerations > 3 m/s^2^ (*n*)	47.0 (21.0)	60.0 (29.0)	48.0 (17.0)	36.0 (7.0)	**0.035**
Decelerations > 3 m/s^2^ (*n*)	44.0 (19.0)	57.0 (26.0)	44.0 (15.0)	24.5 (8.0)	**0.001**
HML distance (m)	963.5 (542.1)	1114.8 (319.9)	981.2 (333.8)	558.6 (41.9)	**0.004**
RPE (au)	3.0 (2.5)	5.0 (1.5)	3.0 (1.0)	2.0 (1.0)	**< 0.001**

Values are presented as median (interquartile range). Athletes were categorized into three groups according to the training session performed: high-load (HL, *n* = 10), moderate-load (ML, *n* = 11), and low-load (LL, *n* = 6). The table reports external and internal training load variables measured during the session. Between-group comparisons were performed using the Kruskal–Wallis test, and p-values refer to overall differences across the three training groups. Given the multifactorial nature of football training sessions, load variables do not necessarily follow a strictly linear gradient across HL, ML, and LL conditions; therefore, results should be interpreted considering both external and internal load indicators

Bold values indicate statistical significance (*p* < 0.05)

BMI, body mass index; HML, high metabolic load; AU, arbitrary unit

Overall, LL sessions were consistently characterized by lower external load, including reduced total distance, speed intensity, dynamic stress load, number of accelerations and decelerations, and high metabolic load distance, compared with ML and HL sessions (all *p* ≤ 0.035).

In contrast, differences between HL and ML sessions were less uniform, with some variables showing comparable values across these two conditions. For example, while HL sessions generally exhibited higher median values for dynamic stress load and heart rate exertion, other parameters, such as high-speed running distance, showed greater variability and partial overlap between ML and HL groups. These findings indicate that training load classification does not follow a strictly linear gradient across all metrics, reflecting the complex and multidimensional nature of football training sessions.

Internal load measures showed a clearer pattern, with HL sessions eliciting the highest cardiovascular and perceptual responses, as indicated by heart rate exertion and RPE (median RPE 5.0), whereas LL sessions were associated with the lowest internal load (median RPE 2.0; *p* < 0.001). ML sessions consistently showed intermediate values.

The results obtained suggest that training-load classification is reflected more consistently by internal load indicators (e.g., RPE) than by individual external load variables, supporting the complementary use of perceived exertion to interpret endocrine responses.

### Steroidomic Response to Training (Pre–Post Analysis)

**Fig. 1:**
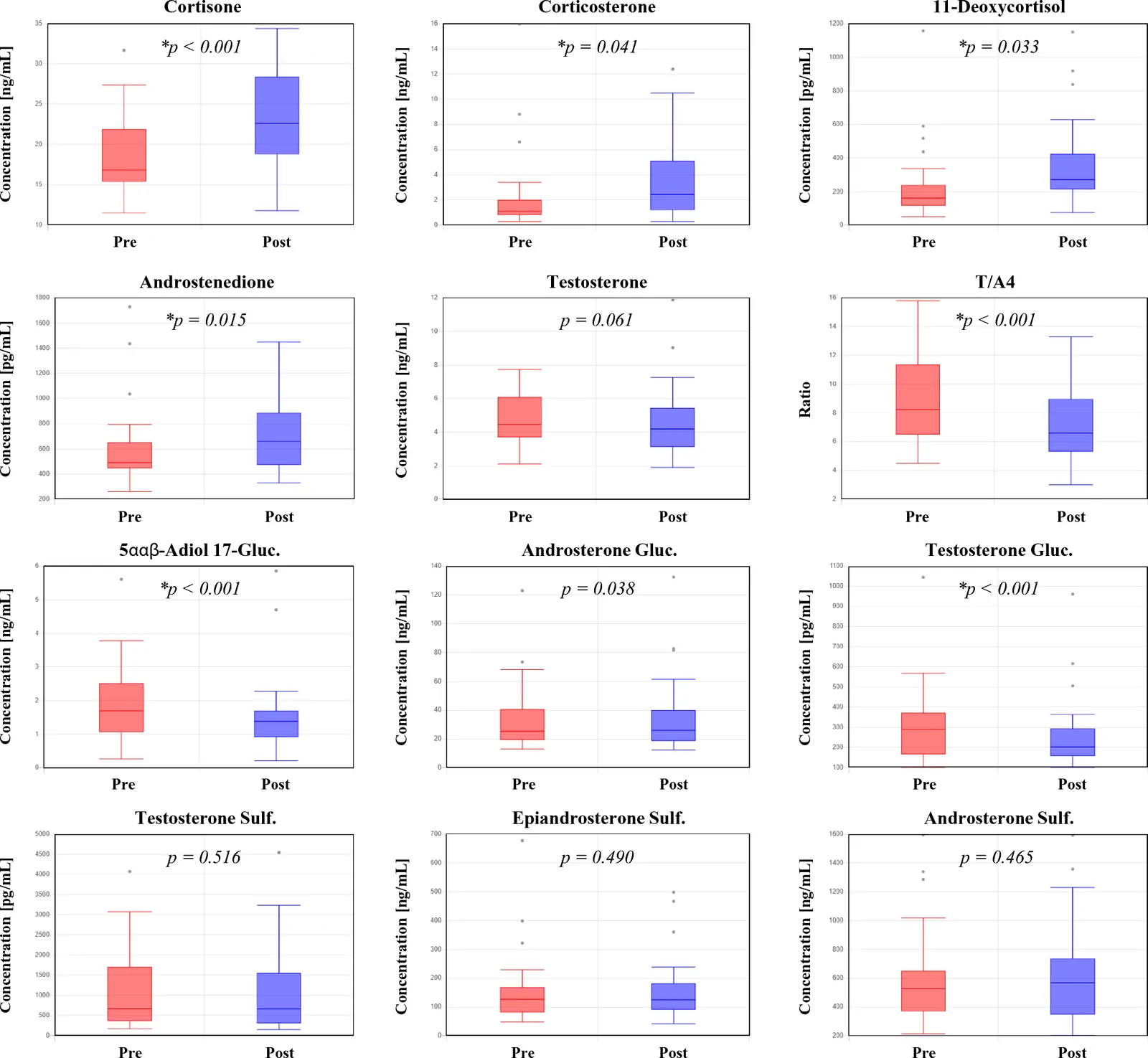
Pre- to post-training changes in selected serum steroids. Box plots show concentrations before (pre, red) and after (post, blue) training for selected circulating steroids, including glucocorticoids, androgens, and phase II metabolites. Each plot represents an individual analyte measured before and after a single training session. Values are in their original measurement units (ng/mL or pg/mL, depending on the analyte), while the testosterone-to-androstenedione ratio (T/A4) is dimensionless. Comparisons should therefore be made within each analyte (pre versus post) rather than across different steroids. The box represents the interquartile range (IQR), the horizontal line indicates the median, whiskers represent 1.5 × IQR, and dots indicate outliers

Among the 26 serum steroids analyzed, 21-deoxycortisol, 11-deoxycorticosterone, epitestosterone, and progesterone were frequently below the lower limit of quantification and were therefore not included in the analysis. Several other steroids, however, exhibited significant pre- to post-training changes (Table 2). Key pre- to post-training changes in selected representative steroids are shown in Fig. 1.

**Table 2:** Pre- and post-training serum steroid concentrations in elite football players

Steroids	Pre-training (*n* = 27)	Post-training (*n* = 27)	*p*-value
Cortisone (ng/mL)	16.9 (6.8)	22.7 (9.9)	**< 0.001**
Cortisol (ng/mL)	79.8 (36.8)	96.4 (55.3	0.253
Corticosterone (pg/mL)	1103.6 (1170.4)	2485.1 (4076.4)	**0.041**
11-Deoxycortisol (pg/mL)	161.3 (134.8)	272.3 (213.5)	**0.033**
Androstenedione (pg/mL)	491.8 (205.8)	659.3 (411.9)	**0.015**
Testosterone (pg/mL)	4462.0 (2403.5)	4183.5 (2332.0)	0.061
DHEA (pg/mL)	1971.6 (1241.5)	3142.6 (2852.3)	**0.003**
17α-Hydroxyprogesterone (pg/mL)	672.2 (505.7)	614.4 (570.4)	0.393
Dihydrotestosterone (pg/mL)	213.3 (175.8)	206.2 (133.1)	0.339
5α-Androstan-3α,17β-diol 3-glucuronide (pg/mL)	743.7 (590.5)	737.0 (708.3)	0.198
5β-Androstan-3α,17β-diol 3-glucuronide (pg/mL)	437.2 (427.7)	346.8 (397.2)	**< 0.001**
5α-Androstan-3α,17β-diol 17-glucuronide (pg/mL)	1694.2 (1508.0)	1373.2 (784.0)	**< 0.001**
5β-Androstan-3α,17β-diol 17-glucuronide (pg/mL)	3598.0 (6859.1)	2673.3 (4718.6)	**< 0.001**
Etiocholanolone glucuronide (ng/mL)	23.6 (15.0)	23.3 (14.3)	0.761
Androsterone glucuronide (ng/mL)	25.4 (21.0)	25.9 (21.8)	**0.038**
Testosterone glucuronide (pg/mL)	290.3 (222.5)	201.7 (138.7)	**< 0.001**
DHEAS (ng/mL)	2408.6 (1019.0)	2452.4 (582.9)	**0.038**
Testosterone sulfate (pg/mL)	671.4 (1398.5)	668.4 (1241.9)	0.516
Epitestosterone sulfate (pg/mL)	369.0 (253.2)	378.1 (200.3)	0.198
Epiandrosterone sulfate (ng/mL)	126.7 (85.4)	125.1 (90.1)	0.490
Etiocholanolone sulfate (ng/mL)	15.9 (38.4)	12.8 (35.3)	**0.002**
Androsterone sulfate (ng/mL)	526.4 (283.5)	569.2 (399.2)	0.465
T/A4	8.2 (4.9)	6.6 (3.7)	**< 0.001**

Steroid concentrations were measured before (pre-training) and after (post-training) a single training session in 26 elite male football players. Values are expressed as median (interquartile range). Paired comparisons were performed using the Wilcoxon signed-rank test, owing to non-normal distribution of the data

Bold values indicate statistical significance (*p* < 0.05)

T/A4, testosterone-to-androstenedione ratio

Following training, free glucocorticoids and unconjugated androgens increased significantly, including cortisone (*p* < 0.001), corticosterone (*p* = 0.041), 11-deoxycortisol (*p* = 0.033), androstenedione (*p* = 0.015), and dehydroepiandrosterone (DHEA) (*p* = 0.003). Among conjugated steroids, multiple androgen glucuronides decreased after training, including 5α-androstan-3α,17β-diol 17-glucuronide, 5β-androstan-3α,17β-diol 17-glucuronide, 5β-androstan-3α,17β-diol 3-glucuronide, androsterone glucuronide, and testosterone glucuronide (all *p* < 0.05). In contrast, dehydroepiandrosterone sulfate (DHEAS) showed a small but significant increase (*p* = 0.038). As shown in Fig. 1, major glucocorticoids and androstenedione increased after training, whereas T/A4 ratio and glucuronidated metabolites tended to decrease. In contrast, most sulfated steroids exhibited limited short-term variation, highlighting their relative stability compared with other steroid fractions.

Consistent with these changes, the testosterone-to-androstenedione ratio (T/A4) decreased significantly following training (median 8.2 to 6.6, *p* < 0.001), indicating an acute shift in circulating androgen balance and potentially compromising its use as a biomarker in the ABP. Other sulfated steroids, including androsterone sulfate, etiocholanolone sulfate, epiandrosterone sulfate, testosterone sulfate, and epitestosterone sulfate, did not show significant pre- to post-training changes. Overall, sulfated steroids displayed a more stable pattern compared with unconjugated and glucuronidated androgens. Comparing changes in serum steroid concentrations (Δ, calculated as post-training minus pretraining values) across training modalities, glucocorticoids showed the most marked load-related responses (Supplementary Table S3). In particular, 11-deoxycortisol exhibited significantly larger post-training increases following HL sessions compared with ML and LL sessions (*p* = 0.037). Cortisol and corticosterone displayed a similar directional pattern, with greater median increases after HL training, although between-group differences for these steroids did not reach statistical significance.

### Response by Training Type

When stratified by training type, HL sessions elicited the most pronounced acute steroidomic responses, whereas ML sessions showed fewer and generally smaller changes, and LL sessions induced minimal or no significant hormonal alterations (Table 3). Following HL training, significant post-training increases were observed in multiple glucocorticoids and unconjugated androgens, including cortisone, corticosterone, 11-deoxycortisol, androstenedione, DHEA, and dihydrotestosterone (all *p* < 0.05). In contrast, several androgen conjugates decreased after HL sessions, including testosterone glucuronide and selected androstanediol glucuronides, alongside a reduction in circulating testosterone. Consistent with these changes, the T/A4 ratio declined after HL training (*p* = 0.004). ML sessions were generally associated with intermediate responses, with some steroids showing directional changes similar to HL but without reaching statistical significance, whereas no significant pre–post changes were detected following LL training, indicating a negligible acute endocrine response to low training load.

**Table 3:** Pre- and post-training serum steroid concentrations by training type

Steroids	High-load (*n* = 10)			Moderate-load (*n* = 11)			Low-load (*n* = 6)		
	Pre-training	Post-training	*p*-value	Pre-training	Post-training	*p*-value	Pre-training	Post-training	*p*-value
Cortisone (ng/mL)	20.0 (4.2)	28.5 (3.4)	**0.008**	16.0 (5.1)	21.5 (8.8)	**0.019**	16.5 (6.3)	17.5 (14.2)	0.563
Cortisol (ng/mL)	85.5 (30.3)	128.8 (12.2)	**0.039**	80.0 (57.8)	90.5 (53.3)	0.638	80.6 (66.1)	66.9 (41.5)	0.313
Corticosterone (pg/mL)	872.2 (847.7)	6223.4 (3337.9)	**0.008**	1282.8 (2964.5)	2538.8 (2912.2)	0.413	1315.9 (2306.4)	1157.1 (565.5)	0.438
11-Deoxycortisol (pg/mL)	147.7 (57.5)	453.1 (256.3)	**0.004**	164.8 (143.0)	253.1 (191.5)	0.320	226.0 (187.6)	216.2 (227.2)	0.438
Androstenedione (pg/mL)	475.0 (137.7)	668.3 (274.3)	**0.008**	508.7 (139.7)	779.2 (661.8)	0.148	602.3 (382.7)	610.7 (812.9)	1.000
Testosterone (pg/mL)	4333.1 (1729.2)	4100.6 (2201.4)	**0.008**	4029.1 (2569.6)	4395.0 (2451.7)	0.465	4803.4 (2557.1)	4611.002 (2731.2)	1.000
DHEA (pg/mL)	1905.6 (228.4)	3535.3 (1480.1)	**0.004**	2165.6 (1217.2)	3436.6 (3778.3)	0.102	1509.2 (2508.3)	1301.5 (2258.1)	1.000
17α-Hydroxyprogesterone (pg/mL)	583.6 (556.9)	625.5 (280.1)	0.820	680.0 (293.3)	627.9 (490.0)	0.831	1042.9 (910.9)	873.4 (1248.4)	1.000
Dihydrotestosterone (pg/mL)	154.1 (108.3)	192.6 (79.0)	**0.027**	185.3 (180.5)	188.4 (139.2)	0.413	217.0 (142.1)	226.7 (206.9)	0.688
5α-Androstan-3α,17β-diol 3-glucuronide (pg/mL)	638.2 (261.1)	766.3 (458.7)	0.203	849.8 (791.4)	946.2 (927.3)	0.700	536.7 (264.6)	561.6 (163.5)	1.000
5β-Androstan-3α,17β-diol 3-glucuronide (pg/mL)	361.2 (210.1)	342.0 (296.3)	0.203	437.5 (444.7)	351.6 (467.3)	**0.010**	455.0 (344.8)	321.9 (371.5)	0.031
5α-Androstan-3α,17β-diol 17-glucuronide (pg/mL)	1021.4 (844.2)	792.3 (882.8)	**0.027**	2395.5 (1192.8)	1617.2 (751.2)	**0.005**	1675.6 (662.5)	1373.2 (418.7)	0.688
5β-Androstan-3α,17β-diol 17-glucuronide (pg/mL)	3591.4 (1150.9)	2306.0 (1338.6)	**0.008**	3000.6 (6959.2)	2093.1 (4731.1)	**0.001**	3165.4 (6158.2)	2361.8 (4552.2)	0.063
Etiocholanolone glucuronide (ng/mL)	23.1 (15.6)	24.9 (14.1)	0.164	23.7 (11.7)	23.3 (12.7)	0.465	22.8 (18.3)	17.4 (20.9)	0.688
Androsterone glucuronide (ng/mL)	19.7 (14.4)	23.0 (18.2)	0.074	29.8 (26.7)	30.3 (30.7)	0.413	19.7 (5.1)	18.0 (11.5)	1.000
Testosterone glucuronide (pg/mL)	281.4 (135.2)	197.6 (102.1)	**0.016**	241.7 (283.3)	177.2 (201.5)	**0.031**	362.4 (174.9)	248.3 (164.1)	0.094
DHEAS (ng/mL)	2438.2 (679.1)	2526.2 (511.1)	0.301	2255.8 (1145.1)	2410.9 (805.3)	0.413	2111.4 (745.2)	2190.4 (624.3)	0.156
Testosterone sulfate (pg/mL)	777.0 (1091.6)	955.7 (1137.6)	0.426	512.4 (687.0)	551.5 (556.8)	0.520	1298.2 (2359.0)	1658.4 (2703.9)	0.688
Epitestosterone sulfate (pg/mL)	304.6 (294.8)	411.0 (147.1)	0.129	314.2 (145.7)	333.3 (249.6)	0.240	471.8 (164.4)	468.7 (227.3)	1.000
Epiandrosterone sulfate (ng/mL)	150.9 (85.5)	135.2 (82.7)	0.359	126.8 (85.1)	115.0 (88.7)	0.765	97.6 (108.3)	111.7 (142.3)	1.000
Etiocholanolone sulfate (ng/mL)	12.8 (0.00)	14.4 (39.6)	0.340	12.8 (0.00)	28.0 (43.3)	0.082	12.8 (0.000)	15.9 (31.8)	0.438
Androsterone sulfate (ng/mL)	464.6 (271.9)	422.8 (331.8)	0.570	578.5 (398.8)	565.0 (392.245)	0.365	526.4 (321.7)	623.2 (533.0)	0.438
T/A4	8.4 (5.864)	5.8 (2.3)	**0.004**	7.1 (5.9)	6.6 (4.5)	0.067	7.4 (4.4)	7.2 (3.5)	1.000

Steroid concentrations are shown for elite football players before and after the three different training sessions: high-load training (HL, *n* = 10), moderate-load (ML, *n* = 11), and low-load (LL, *n* = 6). Values are presented as median (interquartile range). Pre–post comparisons within each group were performed using the Wilcoxon signed-rank test

Bold values indicate statistical significance (*p* < 0.05)

T/A4, testosterone-androstenedione ratio

Between-group comparisons further supported an intensity-dependent pattern of steroidomic responses. In particular, the post-training increase in 11-deoxycortisol was significantly greater after HL than LL sessions (*p* = 0.037), while corticosterone displayed a similar trend without reaching statistical significance.

### Stratification by RPE

Participants were stratified according to session RPE, resulting in RPE < 4 (*n* = 16) and RPE ≥ 4 (*n* = 11) groups. Players reporting RPE ≥ 4 exhibited higher external and internal training loads, accompanied by larger acute endocrine response (Supplementary Table S4).

Specifically, post-training increases (Δ) in cortisol, corticosterone, 11-deoxycortisol, and DHEA were significantly greater in the RPE ≥ 4 group compared with the RPE < 4 group (all *p* ≤ 0.013), indicating a close association between perceived exertion and activation of glucocorticoid and androgen pathways.

In multivariable logistic regression models adjusted for training type, heart rate exertion, dynamic stress load, and speed intensity, greater post-training increases in cortisol (OR 0.956, *p* = 0.032), 11-deoxycortisol (OR 0.990, *p* = 0.010), and DHEA (OR 0.999, *p* = 0.049) were independently associated with a higher likelihood of reporting RPE ≥ 4 (Supplementary Table S5). Corticosterone showed a similar trend without reaching conventional statistical significance (*p* = 0.060).

### Mixed-Effects Model Results

Linear mixed-effects models were used to evaluate acute changes in serum steroid concentrations while accounting for training modality and load-related covariates (Table 4). Models included fixed effects for time (post- versus pretraining), training type (LL, ML, HL), and their interactions, with player identity specified as a random effect. RPE, dynamic stress load, speed intensity, and heart rate exertion were included as covariates.

**Table 4:** Linear mixed models for the effect of training and load parameters on serum steroid levels

Steroids	Post- versus pretraining			Pre-training						Time × moderate-load			Time × high-load		Significan*t* covariates		Model *p*-value
				Moderate-load versus low-load			High-load versus low-load										
	Coeff	*p*-value	Coeff		*p*-value	Coeff		*p*-value	Coeff		*p*-value	Coeff		*p*-value			
Cortisone (ng/mL)	1.841	0.335	− 2.209		0.561	1.462		0.806	1.653		0.486	5500		**0.026**			**0.003**
Androstenedione (pg/mL)	39.983	0.708	− 40.641		0.867	169.839		0.658	76.779		0.564	167.209		0.226			**< 0.001**
Testosterone (pg/mL)	636.264	0.223	1424.508		0.310	3519.13		0.114	− 747.521		0.249	− 1153.423		0.087			**< 0.001**
DHEA (pg/mL)	− 32.012	0.966	1529.752		0.259	710.694		0.736	1274.996		0.168	1679.219		0.080			**0.010**
17α-Hydroxyprogesterone (pg/mL)	19.601	0.886	− 210.452		0.558	− 7.819		0.989	− 26,534		0.876	− 46.191		0.794			**< 0.001**
Dihydrotestosterone (pg/mL)	44.137	0.069	127.420		0.097	186.172		0.128	− 56.414		0.062	− 18.696		0.551			**< 0.001**
5α-Androstan-3α,17β-diol 3-glucuronide (pg/mL)	− 10.375	0.877	520.766		0.123	187.502		0.730	15.834		0.849	104.462		0.227			**< 0.001**
5β-Androstan-3α,17β-diol 3-glucuronide (pg/mL)	− 78.346	0.315	531.620		0.061	1056.289		**0.020**	− 68.924		0.477	− 3.832		0.970	RPE		**< 0.001**
5α-Androstan-3α,17β-diol 17-glucuronide (pg/mL)	− 9.767	0.955	1750.919		**0.046**	684.774		0.626	− 543.035		**0.013**	− 226.342		0.316			**< 0.001**
5β-Androstan-3α,17β-diol 17-glucuronide (pg/mL)	− 739.5	0.262	3945.417		0.190	8838.538		0.067	− 728.729		0.374	− 612.589		0.472			**< 0.001**
Etiocholanolone glucuronide (ng/mL)	− 680.662	0.574	14,959.52		0.123	29,236.27		0.061	− 431.791		0.774	1817.791		0.245			**< 0.001**
Androsterone glucuronide (ng/mL)	395.794	0.855	18,807.78		0.112	− 1472.163		0.938	893.623		0.739	3002.935		0.281			**< 0.001**
Testosterone glucuronide (pg/mL)	− 52.231	**0.028**	35.907		0.823	144.486		0.575	− 4.663		0.875	18.961		0.537			**< 0.001**
DHEAS (ng/mL)	277.404	0.043	424.487		0.518	548.899		0.602	− 294.792		0.084	− 114.779		0.517			**< 0.001**
Testosterone sulfate (pg/mL)	194.503	0.135	− 725.431		0.135	− 45.583		0.975	− 207.149		0.200	− 259.406		0.123			**< 0.001**
Epitestosterone sulfate (pg/mL)	6.046	0.907	− 85.644		0.814	144.836		0.804	30.484		0.636	35.356		0.597			**< 0.001**
Epiandrosterone sulfate (ng/mL)	11.159	0.317	− 5.048		0.945	− 4.182		0.972	− 5.261		0.705	− 5.315		0.712			**< 0.001**
Androsterone sulfate (ng/mL)	73.732	**0.014**	− 89.487		0.721	− 114.182		0.777	− 85.144		**0.023**	− 56.601		0.146			**< 0.001**
T/A4	0.066	0.146	3.423		0.114	4.370		0.201	− 2.004		0.091	− 3.648		**0.003**			**< 0.001**

Results from linear mixed-effects models evaluating the effect of training on serum steroid concentrations in elite football players. Fixed effects included time (post versus pre-training), training type (LL, ML, HL), and their interactions (time × ML; time × HL). Each model was adjusted for relevant covariates, including heart rate exertion, dynamic stress load, speed intensity, and rating of perceived exertion (RPE). Coefficients represent estimated changes in steroid levels associated with each fixed effect. Model fit is reported as marginal *R*^2^ where available

Bold values indicate statistical significance (*p* < 0.05)

RPE, rating of perceived exertion; T/A4, testosterone-androstenedione ratio

Among glucocorticoids, 11-deoxycortisol and corticosterone showed significant training-dependent responses, with positive time × HL interactions indicating greater post-training increases following HL sessions compared with LL sessions (11-deoxycortisol: *β* =  + 479.6 pg/mL, *p* = 0.010; corticosterone: *β* =  + 5906.4 pg/mL, *p* = 0.021). Cortisol exhibited a similar direction of effect but did not reach statistical significance in the mixed-effects models. Regarding androgens, testosterone glucuronide decreased significantly over time, independent of training modality (*β* =  − 52.2 pg/mL, *p* = 0.028), while DHEAS showed a modest post-training increase (*β* =  + 277.4 ng/mL, *p* = 0.043). The T/A4 ratio declined significantly following HL training, as reflected by a negative time × HL interaction (*β* =  − 3.6, *p* = 0.003). Several phase II androgen metabolites also displayed training- or load-associated modulation, including significant interactions for 5β-androstan-3α,17β-diol 3-glucuronide and 5α-androstan-3α,17β-diol 17-glucuronide.

For steroids showing suboptimal mixed-model fit, complementary linear regression analyses were performed using the same predictors and covariates (Supplementary Table S2). These analyses yielded results consistent with the mixed-effects models, confirming a marked HL–dependent increase in 11-deoxycortisol and supporting a training-intensity-dependent response for cortisol. Etiocholanolone sulfate did not show significant training-related effects in the mixed-effects models, although a borderline association with perceived exertion was observed in complementary analyses. Sulfated steroids showed limited sensitivity to training modality and load-related covariates, with the exception of DHEAS, which exhibited a modest but consistent post-training increase.

## Discussion

### Training Load, Perceived Exertion and Acute Steroid Regulation

Acute endocrine response to football training varied systematically according to training load. Free glucocorticoids, including cortisone, corticosterone, and 11-deoxycortisol, together with unconjugated androgens such as androstenedione, DHEA, and dihydrotestosterone, increased following training, with the largest changes observed after high-load sessions. This response pattern aligns with activation of the HPA axis and stimulation of steroidogenesis during high-intensity exercise, as documented across endurance and team-sport settings [4–7]. The progressive scaling of hormonal responses from low- to moderate- and high-load sessions further supports a close relationship between exercise intensity and short-term endocrine regulation [4, 8]. Beyond absolute hormone concentrations, training also altered the relative balance between key androgenic pathways. In particular, the T/A4 ratio declined following ML and HL sessions, suggesting a transient shift in androgen precursor–product dynamics. Such alterations may reflect exercise-induced changes in steroidogenic flux or increased peripheral metabolism of testosterone during acute physiological stress. Comparable reductions in T/A4 and T/F ratios have been linked to training stress, recovery status, and catabolic–anabolic balance in athletes [4, 5, 35], supporting the relevance of these ratios as indicators of acute training strain.

From a physiological perspective, these endocrine responses can be interpreted in relation to the specific characteristics of the training stimulus. High-load sessions in football typically involve a greater volume of high-intensity running, repeated accelerations and decelerations, and elevated cardiovascular strain, all of which are known to increase metabolic and neuroendocrine stress. Such stimuli are closely associated with activation of the HPA axis and increased glucocorticoid secretion, as well as modulation of androgen metabolism. In this context, external load metrics (e.g., high metabolic load distance, speed intensity) and internal load indicators (e.g., heart rate exertion and perceived exertion) provide complementary information to interpret the observed steroid responses in relation to the underlying physiological demands of training.

Concomitantly, several glucuronidated androgen metabolites decreased after training, particularly following HL sessions. This pattern is consistent with acute modulation of androgen clearance pathways under conditions of elevated physiological demand, as previously reported after endurance- and sprint-based exercise [3, 5, 36]. The parallel increase in unconjugated androgens and decrease in glucuronidated metabolites points to coordinated regulation across steroid biosynthesis and metabolism, rather than isolated changes in individual hormone pools.

Internal training load further modulated endocrine responses. When stratified by perceived exertion, players reporting higher RPE values exhibited larger post-training increases in glucocorticoids and androgens, including cortisol, 11-deoxycortisol, and DHEA. These associations persisted after adjustment for objective load metrics, indicating that perceived exertion captures aspects of physiological stress not fully reflected by external load alone [8, 37]. This is particularly relevant in football, where perceived exertion integrates multiple physiological and psychological stressors, providing a practical proxy of the overall internal load experienced by the player. Similar relationships between internal load, endocrine perturbations, and recovery dynamics have been described in elite football contexts, underscoring the interpretative value of subjective load measures in applied environments [28, 38].

Multivariable mixed-effects and regression analyses reinforced this multifactorial framework, demonstrating that acute endocrine response were shaped by both training modality and specific load-related covariates, including speed intensity and heart-rate exertion. Together, these observations highlight the complexity of steroid regulation in response to training and the need to integrate external load, internal load, and biochemical markers when interpreting short-term endocrine adaptations in elite football players. Circadian variation should also be considered when interpreting these findings. Blood sampling was performed in the mid-afternoon, a time window corresponding to the descending phase of ACTH-related steroid secretion, when glucocorticoid concentrations are generally lower and progressively declining. This timing was intentionally selected during the study design to reduce the potential confounding influence of circadian peaks. In this context, the post-training increases observed in glucocorticoids and unconjugated androgens, particularly following higher-load sessions, are not consistent with the expected circadian trend alone, which would rather predict stable or decreasing concentrations over this time interval. Therefore, circadian influences may have attenuated, rather than generated, the observed exercise-related responses. Nevertheless, the relative contribution of circadian rhythms and exercise-induced effects cannot be fully disentangled within the present design and should be specifically addressed in future studies.

### Steroid Conjugation Pathways and Short-Term Endocrine Stability

In addition to changes in unconjugated hormones, training sessions induced distinct, load-associated modulation of several phase II androgen metabolites. Multiple glucuronidated androgens, including testosterone glucuronide and androstanediol glucuronide isomers, declined after training, with the most pronounced reductions occurring following high-load sessions. Such responses are compatible with evidence that acute physical stress can influence androgen conjugation and clearance processes [3, 5, 36]. Glucuronidation represents a major route for androgen inactivation and elimination. Short-term reductions in circulating glucuronidated metabolites may therefore reflect transient redistribution of steroid metabolism during acute exercise. Potential mechanisms include altered hepatic conjugation capacity, changes in peripheral metabolism, or shifts in the balance between steroid production and clearance under conditions of elevated physiological demand. The concurrent increase in unconjugated androgens observed here supports the concept of integrated regulation across biosynthetic and metabolic pathways rather than independent modulation of individual steroid fractions.

By contrast, most sulfated steroids displayed relative short-term stability across training conditions. Androgens such as androsterone sulfate, epitestosterone sulfate, and testosterone sulfate showed minimal acute variation irrespective of training load or perceived exertion, consistent with the longer half-life and slower turnover of sulfated steroids compared with unconjugated and glucuronidated forms [23, 24]. This relative stability represents a particularly relevant finding of the present study, as it suggests that sulfated steroids are less sensitive to acute exercise-induced perturbations. From a physiological perspective, this likely reflects their role as more stable circulating reservoirs, characterized by slower metabolic turnover and reduced susceptibility to rapid fluctuations in response to acute stress. DHEAS represented a partial exception, exhibiting a modest but consistent post-training increase, which may reflect acute activation of adrenal steroidogenesis rather than altered clearance, in agreement with previous observations during high-intensity exercise [4, 6].

From an applied perspective, the differential behavior of phase II metabolites may have important implications for the interpretation of circulating steroid profiles. Compared with more labile steroid fractions, sulfated metabolites may provide more robust information in contexts where samples are collected in close temporal proximity to training exposure, as they appear less influenced by short-term physiological stressors. However, given the nonstandardized sampling conditions of the present study, further investigations under controlled and ABP-compliant protocols are required to confirm their potential utility in longitudinal monitoring frameworks.

Acute training appears to differentially affect steroid conjugation pathways, with glucuronidated metabolites showing greater sensitivity to short-term exercise-induced stress and sulfated steroids remaining comparatively resistant to acute perturbation. These divergent behaviors likely reflect differences in metabolic turnover and clearance kinetics across steroid classes and have direct implications for interpretation of circulating steroid profiles collected in close temporal proximity to training exposure.

### Implications for Blood Steroid Profiling and the Athlete Biological Passport

The endocrine patterns observed in this study are directly relevant for the interpretation of BSP variations. It should be noted that current ABP guidelines require a delay between exercise and blood sampling. Therefore, the immediate post-training sampling adopted in this study does not reflect standard ABP conditions and limits direct translation of these findings to antidoping practice. In addition, the present study design does not allow assessment of within-individual variability over time, which represents a key component of BSP interpretation within the ABP framework. As each player was exposed to a single training condition, the observed differences reflect between-subject comparisons and may be influenced by baseline interindividual variability. Therefore, the findings should not be interpreted as directly informing individual longitudinal profiles but rather as providing insight into the magnitude and direction of acute exercise-induced changes. Future studies using repeated-measures designs within the same athletes and standardized sampling conditions are warranted to better characterize within-subject variability and its implications for ABP interpretation. Several BSP components, particularly androstenedione and the T/A4 ratio, exhibited marked sensitivity to training load and perceived exertion, indicating that recent exposure to ML or HL football training constitutes a relevant physiological factor when blood samples are collected shortly after exercise. Exercise-related reductions in the T/A4 ratio illustrate how acute training stress can transiently modify androgen precursor–product balance. Within a longitudinal ABP framework, such short-term fluctuations may contribute to within-athlete variability. These changes should be interpreted in relation to recent training exposure, internal load, and sampling conditions. Without adequate contextualization, physiological responses to exercise could be misclassified as atypical biological variation rather than expected consequences of training.

Phase II metabolites could further help in refining BSP interpretation. While glucuronidated androgens demonstrated clear short-term sensitivity to training load, sulfated steroids remained relatively stable across training conditions. Owing to their slower turnover and reduced susceptibility to acute exercise-induced perturbations, sulfated metabolites may provide more robust information for longitudinal monitoring within the ABP.

From an applied perspective, these considerations emphasize the importance of integrating biochemical steroid profiles with information on recent training load, perceived exertion, and sampling timing. Such contextualization can improve physiological interpretation of BSP fluctuations and enhance discrimination between exercise-related variability and changes of potential antidoping relevance.

### Limitations

Several limitations merit consideration. The study population consisted exclusively of male elite football players from a single professional club, limiting generalizability to female athletes, other competitive levels, or different sporting disciplines. The sampling strategy focused on immediate pre- and post-training measurements, capturing acute endocrine response but not allowing assessment of delayed or recovery-phase hormonal dynamics that may also be relevant for training adaptation. Furthermore, the absence of a standardized post-exercise delay in sampling limits the direct applicability of the results to ABP-compliant testing conditions. Although sampling was performed during the descending phase of circadian steroid secretion, circadian variation, particularly for glucocorticoids, may still have partially influenced the observed changes and cannot be fully separated from exercise-induced effects. In addition, samples were stored at − 20 °C rather than − 80 °C, which is commonly used in long-term metabolomic studies. However, previous studies have shown that circulating steroid hormones in serum are generally stable under − 20 °C storage conditions over short-to-medium timeframes [39]. In the present study, storage duration was limited (maximum 3 months), and precautions were taken to avoid repeated freeze–thaw cycles, which are known to affect steroid measurements [40]. Nevertheless, a potential impact on the stability of some analytes cannot be entirely excluded. Although an extensive blood steroidomic panel was applied, other physiological systems involved in the response to training stress, such as inflammatory, metabolic, or neuromuscular pathways, were not evaluated and may provide complementary information. Psychological factors may also influence endocrine responses; however, their specific contribution could not be directly assessed within the present study design. Even simple interindividual variability may explain between-group differences. In this context, interindividual variability may exceed intra-individual changes, particularly owing to baseline differences between players. This aspect, together with the between-subject design, may influence the interpretation of observed differences across training conditions and further supports the exploratory nature of the findings. In addition, the observational design and nonrandomized allocation of training sessions preclude causal inference regarding the contribution of individual training components. Given the sample size and the number of outcomes assessed, the present findings should be interpreted as exploratory and require confirmation in larger, independent cohorts. Residual variability related to factors such as hydration status, nutritional intake, sleep, or subclinical illness cannot be fully excluded. Finally, although both external and internal loads of the experimental training sessions were quantified, the training load from the preceding day was not considered, despite its potential influence on hormonal responses.

### Conclusions

Football training is associated with acute endocrine adjustments that vary according to training load and perceived exertion. HL sessions were characterized by larger perturbations in circulating glucocorticoids and unconjugated androgens, while phase II metabolites displayed heterogeneous responses, with glucuronidated steroids showing greater sensitivity and sulfated steroids remaining comparatively stable.

The observed sensitivity of androstenedione and the T/A4 ratio to recent training exposure suggests that exercise-related variability should be considered when interpreting BSP data collected in proximity to training.

Taken together, these results highlight the potential value of integrating steroidomic measurements with training-load metrics to improve the physiological interpretation of short-term endocrine variability in elite football players. However, further studies, including repeated-measures designs, different populations, and standardized sampling protocols, are required before translating these observations into applied or antidoping frameworks.

## Supplementary Information

Below is the link to the electronic supplementary material.

Supplementary file1 (PDF 276 KB)

## Declarations

### Availability of Data and Material

The datasets generated and/or analyzed during the current study are available from the corresponding author on reasonable request.
