# Supplementary material for: Acute Effects of Different Training Sessions on the Extended Blood Steroid Profile in Elite Male Football Players
Source: Sports Med. 2026 Jun 2;56(10):2569–82. doi: 10.1007/s40279-026-02467-8 (PMC13615034; doi:10.1007/s40279-026-02467-8)
Supplement: Supplementary file 1 [file 40279_2026_2467_MOESM1_ESM.pdf]

Sports Medicine

Supplementary Material

Acute Effects of Different Training Sessions on the Extended Blood Steroid Profile in Elite Male Football Players

1.1 Training Sessions Characteristics

Players completed one of three training session types, classified as LL, ML or HL sessions based on their planned duration and content. LL sessions consisted of a 10-min warm-up, followed by 15 min of ball possession drills (7 vs 7 with floaters; pitch dimensions: 37 × 40 m) and 25 min of tactical work, for a total duration of 50 min. ML sessions included a 12-min warm-up, 12 min of technical drills, 18 min of tactical work, and 18 min of medium-sided games (10 vs 10 with goalkeepers; pitch dimensions: 68 × 60 m), with a total duration of 60 min. HL sessions comprised a 12-min warm-up, 16 min of ball possession drills (7 vs 7 with floaters; pitch dimensions: 37 × 40 m), 25 min of tactical work, and 18 min of small-sided games (3 vs 3 and 4 vs 4 with goalkeepers; pitch dimensions: 28 × 32 m), resulting in a total duration of 70 min.

Table S1 – **List of unconjugated and phase II steroids quantified by UHPLC–MS/MS**

	Steroids	Abbreviations
Free steroids	**Glucocorticoids**	
	Cortisone, ng/mL	E
	Cortisol, ng/mL	F
	11-Deoxycorticosterone, pg/mL	DOC
	11-Deoxycortisol, pg/mL	11-DF
	21-Deoxycortisol, pg/mL	21-DF
	Corticosterone, pg/mL	CORT
	**Androgens**	
	17α-Hydroxyprogesterone, pg/mL	17αOHP
	Androstenedione, pg/mL	A4
	Dehydroepiandrosterone, ng/mL	DHEA
	Dihydrotestosterone, pg/mL	DHT
	Epitestosterone, pg/mL	EpiT
	Progesterone, pg/mL	P
	Testosterone, pg/mL	T
Glucuronides	5α-androstan-3α,17β-diol 3-glucuronide, pg/mL	5ααβ-Adiol 3G
	5β-androstan-3α,17β-diol 3-glucuronide, pg/mL	5βαβ-Adiol 3G
	5α-androstan-3α,17β-diol 17-glucuronide, pg/mL	5ααβ-Adiol 17G
	5β-androstan-3α,17β-diol 17-glucuronide, pg/mL	5βαβ-Adiol 17G
	Androsterone glucuronide, ng/mL	AG
	Etiocholanolone glucuronide, ng/mL	EtioG
	Testosterone glucuronide, pg/mL	TG
Sulfates	Androsterone sulfate, ng/mL	AS
	Dehydroepiandrosterone sulfate, ng/mL	DHEAS
	Epiandrosterone sulfate, ng/mL	EpiAS
	Epitestosterone sulfate, pg/mL	EpiTS
	Etiocholanolone sulfate, ng/mL	EtioS
	Testosterone sulfate, pg/mL	TS

1.2 Steroid analysis by UHPLC-MS/MS

Serum samples were collected using SST™ II Advance BD Vacutainer tubes (6 mL; Becton, Dickinson & Company, Franklin Lakes, USA). Samples were immediately transported to the laboratory, stabilized at room temperature for 15 minutes, and centrifuged at 1500 g for 15 minutes. Serum aliquots (~500 µL) were stored in 5 mL screw-cap polypropylene tubes (Sarstedt, Nümbrecht, Germany) at –20°C until analysis.

Serum steroids were extracted from 200 µL of sample using solid-phase extraction (SPE) on EVOLUTE® EXPRESS ABN 30 mg 96-well plates (Biotage, Uppsala, Sweden). Prior to extraction, samples were spiked with a mixture of isotopically labelled internal standards and diluted with phosphoric acid (final concentration 2%) to promote protein dissociation. SPE cartridges were conditioned with methanol and equilibrated with aqueous phosphoric acid solution. After sample loading, SPE plate was washed with pure water, then analytes were eluted with a methanol/acetonitrile (1:1, v/v) mixture. Extracts were evaporated under nitrogen at 50 °C and reconstituted in methanol/water (1:1, v/v); 20 µL were injected into the UHPLC-MS/MS system consisting in a Nexera X2 UHPLC system (Shimadzu, Tokyo, Japan) coupled with a Citrine Triple Quad MS/MS detector (AB Sciex, Ontario, Canada). Chromatographic separation was performed using a Kinetex PS C18 column (150 × 2.1 mm, 2.6 µm; Phenomenex, Torrance, CA, USA) maintained at 55 °C. Mobile phase A consisted of 10 mM ammonium acetate in water, and mobile phase B of 10 mM ammonium acetate in methanol. A multistep gradient elution was applied, achieving baseline separation of all target analytes, including isomeric glucuronide and sulfate metabolites, within a total run time of 25 min. The flow rate was set at 300 µL/min.

Mass spectrometric detection was carried out on the triple quadrupole mass spectrometer equipped with an electrospray ionization source operating in polarity-switching mode. Source parameters were optimized to ensure adequate sensitivity across a wide dynamic

concentration range, with source temperature set at 550 °C. Quantification was based on multiple reaction monitoring (MRM), using two transitions (quantifier and qualifier) for unconjugated and glucuronidated steroids, and one transition for sulfated steroids detected in negative ionization mode. Instrumental parameters for each analyte and internal standard were optimized by direct infusion of reference solutions.

The method was fully validated in accordance with ISO/IEC 17025:2017 and World Anti-Doping Agency (WADA) requirements. Validation parameters included selectivity, extraction recovery, matrix effects, linearity, trueness, repeatability, intermediate precision, combined measurement uncertainty, lower limit of quantification (LLOQ), carry-over, robustness, and extract stability. To ensure analytical accuracy, three levels of external quality control samples (MassCheck® Steroid Serum Controls, Chromsystems, Gräfelfing, Germany), containing twelve endogenous steroids and DHEAS, were included in each analytical batch. Extraction recoveries ranged from approximately 85% to 98%, with matrix effects close to unity for all analytes. LLOQs ranged from low pg/mL for unconjugated steroids to low ng/mL or µg/mL for sulfate conjugates, reflecting their physiological concentration ranges. The method demonstrated satisfactory precision and accuracy across the validated concentration ranges and was shown to be robust and suitable for high-throughput analysis of extended blood steroid profiles.

Table S2 – Linear Regression Models for Steroid Responses to Training. Linear regression models were used to assess pre-post changes in selected serum steroids in elite football players. These models were applied when linear mixed-effects models showed inferior fit or convergence issues. Independent variables included training type (HL, ML, LL), training-time interactions, and training load covariates (RPE, speed intensity). Coefficients represent estimated differences in steroid concentration. Significant covariates with p <0.15 are reported in the last column. Abbreviations: HL, high-load training; ML, moderate-load training; LL, low-load training; RPE, rating of perceived exertion; Time × ML, interaction term for time and ML training; Time × HL, interaction term for time and HL training.

Steroids	Post vs Pre-training		Pre-training ML vs LL		Pre-training HL vs LL		Time x ML		Time x HL		Significant Covariates
	Coeff	p-value	Coeff	p-value	Coeff	p-value	Coeff	p-value	Coeff	p-value	
**11-DF (pg/mL)**	-135.873	0.330	-318.005	0.070	-486.176	0.063	272.772	0.119	479.550	0.010	Speed Int. (p=0.097)
**F (ng/mL)**	-26.521	0.230	-10.575	0.698	-25.931	0.523	33.511	0.223	69.066	0.018	
**CORT (pg/mL)**	-1490.746	0.439	-1385.04	0.562	-5004.315	0.163	2109.429	0.163	5906.43	0.021	
**EtioS (ng/mL)**	17.381	0.315	-2.469	0.908	26.100	0.413	14.933	0.486	-3.273	0.883	RPE (p=0.058)

Table S3 - Pre- to post-training changes (Δ) in serum steroid concentrations according to training modality. Values are presented as median (interquartile range). Changes (Δ) were calculated as post-training minus pre-training concentrations; therefore, positive values indicate an increase after training, whereas negative values indicate a decrease. Athletes were categorized into three groups according to the training session performed: high-load (HL, N = 10), moderate-load (ML, N = 11), and low-load (LL, N = 6). Between-group comparisons were performed using the Kruskal–Wallis test, and p-values refer to overall differences across training conditions.

Variables/Parameters	Total (N=27)	High-load (N=10)	Moderate-load (N=11)	Low-load (N=6)	p-value
**Δ Cortisone (ng/mL)**	3.6 (8.4)	7.4 (6.1)	1.4 (6.3)	1.0 (7.2)	0.174
**Δ Cortisol (ng/mL)**	6.8 (81.5)	29.5 (67.2)	-0.8 (85.0)	-12.2 (67.5)	0.078
**Δ Corticosterone (pg/mL)**	321.7 (4858.7)	4447.8 (5860.5)	86.2 (3954.6)	-183.1 (3079.2)	0.064
**Δ 11-Deoxycortisol (pg/mL)**	147.0 (299.6)	263.3 (349.6)	147.0 (299.6)	-63.1 (319.8)	**0.037**
**Δ Androstenedione (pg/mL)**	112.0 (309.9)	131.3 (212.1)	77.7 (356.4)	-18.8 (327.4)	0.544
**Δ Testosterone (pg/mL)**	-417.7 (1416.6)	-344.7 (568.2)	-576.3 (1513.6)	-55.4 (1939.2)	0.339
**Δ DHEA (pg/mL)**	867.6 (2363.8)	1501.8 (1597.2)	680.1 (2762.8)	13.6 (1404.4)	0.254
**Δ 17α-Hydroxyprogesterone (pg/mL)**	-10.5 (283.9)	-23.1 (184.9)	-7.3 (283.9)	-76.9 (450.8)	0.970
**Δ Dihydrotestosterone (pg/mL)**	7.1 (57.5)	22.8 (27.9)	-15.1 (53.8)	17.8 (43.5)	0.178
**Δ 5α-androstan-3α,17β-diol 3-glucuronide (pg/mL)**	50.8 (262.6)	161.3 (224.1)	31.3 (285.0)	-59.7 (268.5)	0.289
**Δ 5β-androstan-3α,17β-diol 3-glucuronide (pg/mL)**	-48.0 (150.7)	-55.9 (233.2)	-48.0 (179.0)	-68.6 (113.9)	0.782
**Δ 5α-androstan-3α,17β-diol 17-glucuronide (pg/mL)**	-236.8 (409.0)	-214.3 (311.7)	-426.6 (653.7)	-186.5 (510.3)	0.067
**Δ 5β-androstan-3α,17β-diol 17-glucuronide (pg/mL)**	-800.8 (1602.1)	-1144.8 (1329.7)	-386.9 (2238.1)	-730.9 (698.8)	0.673
**Δ Etiocholanolone Glucuronide (ng/mL)**	-20109.8 (14488.7)	-20663.9 (15394.9)	-20171.0 (644.0)	-13895.0 (804.0)	0.708
**Δ Androsterone Glucuronide (ng/mL)**	1622.1 (8883.1)	3652.8 (9127.0)	1625.3 (9210.4)	-1640.7 (5933.5)	0.328
**Δ Testosterone Glucuronide (pg/mL)**	-39.2 (81.8)	-35.1 (47.0)	-64.5 (84.1)	-54.8 (70.6)	0.664
**Δ DHEAS (ng/mL)**	98.0 (291.3)	122.7 (324.1)	47.9 (289.7)	162.6 (414.7)	0.239
**Δ Testosterone Sulfate (pg/mL)**	1508.3 (1343.1)	2102.1 (1430.2)	1600.6 (1146.3)	1158.0 (1741.4)	0.139
**Δ Epitestosterone Sulfate (pg/mL)**	44.2 (140.8)	66.2 (72.8)	20.6 (182.2)	-4.8 (156.4)	0.867
**Δ Epiandrosterone Sulfate (ng/mL)**	2.2 (18.2)	3.6 (11.4)	0.6 (19.5)	-3.0 (43.6)	0.929

**Δ Etiocholanolone Sulfate (ng/mL)**	-1.7 (6.9)	-0.7 (6.9)	-4.2 (7.2)	-1.1 (7.5)	0.193
**Δ Androsterone Sulfate (ng/mL)**	1.7 (58.8)	12.1 (69.8)	-4.7 (58.3)	17.3 (204.9)	0.115
**Δ T/A4**	-1.9 (3.5)	-3.5 (4.1)	-2.0 (3.4)	-0.2 (1.9)	0.053

Table S4 - Training Load Parameters and Steroidomic Changes Stratified by rating of perceived exertion (RPE). Players were divided into two groups based on session RPE: RPE <4 (N = 16) and RPE ≥4 (N = 11). The table reports anthropometric data, external and internal load variables, and changes in serum steroid concentrations (Δ) between pre- and post-training. Values are presented as median (interquartile range). Group comparisons were performed using the Mann-Whitney U test. Abbreviations: BMI, body mass index; Δ, delta (post minus pre values); DSL, dynamic stress load; Explosive Distance, distance covered at high acceleration thresholds; HML, high metabolic load; HMLD, high metabolic load distance; RPE, rating of perceived exertion; au, arbitrary unit.

Variables/Parameters	Total (N=27)	RPE <4 (N=16)	RPE ≥4 (N=11)	p-value
**Age (years)**	19 (2)	19 (2)	19 (1)	0.890
**Weight (Kg)**	76 (8)	77.4 (8.6)	74.4 (4.3)	0.257
**BMI (Kg/m^2)**	22.800 (2.4)	22.7 (2.0)	23.5 (1.8)	0.090
**Total Distance (m)**	6098.7 (1509.4)	5287.7 (1839.6)	6531.9 (711.4)	< 0.001
**Distance > 20 km/h (m)**	150.1 (160.9)	117.6 (133.8)	241.3 (127.9)	0.007
**Distance > 25 km/h (m)**	9.9 (20.2)	4.2 (19.1)	12.8 (18.8)	0.233
**Max Speed (km/h)**	26.8 (3.6)	26.2 (5.4)	27.2 (2.4)	0.280
**Speed Intensity (au)**	292.2 (75.6)	250.045 (95.4)	315.920 (34.5)	< 0.001
**Max Heart Rate (bpm)**	183.0 (20.4)	177 (14)	192 (12)	0.009
**Average Heart Rate (bpm)**	137.3 (26.1)	134 (19.8)	146.170 (20.4)	0.078
**Time > 85% HR max (%)**	6.5 (24.7)	3.2 (11.9)	14.090 (24.9)	0.101
**Heart Rate Exertion (au)**	135.8 (121.7)	124.1 (60.2)	175.640 (125.7)	0.011
**Dynamic Stress Load (au)**	212.4 (193.9)	121.3 (130.9)	291.810 (140.3)	0.005
**Accelerations > 3 m/s^2 (n)**	47 (21)	39 (17)	59 (29)	0.001
**Decelerations > 3 m/s^2 (n)**	44 (19)	36 (20)	52 (26)	< 0.001
**HML Distance (m)**	963.5 (542.4)	734.8 (370.6)	1186.4 (289.3)	< 0.001
**RPE (au)**	3.0 (2.5)	3.0 (0.8)	5.0 (1.5)	< 0.001
**Δ Cortisone (ng/mL)**	3.6 (8.5)	1.3 (6.4)	6.3 (6.4)	0.062
**Δ Cortisol (ng/mL)**	6.8 (81.5)	-4.5 (37.2)	64.1 (53.8)	0.003
**Δ Corticosterone (pg/mL)**	321.7 (4858.7)	46.9 (2026.3)	5829.4 (4943.7)	< 0.001
**Δ 11-Deoxycortisol (pg/mL)**	147.0 (299.6)	-9.7 (319.7)	285.2 (495.2)	< 0.001
**Δ Androstenedione (pg/mL)**	110.0 (309.9)	39.3 (246.5)	234.3 (296.5)	0.082
**Δ Testosterone (pg/mL)**	-417.7 (1416.6)	-171.1 (1556.0)	-587.7 (844.3)	0.104
**Δ DHEA (pg/mL)**	867.6 (2363.8)	230.5 (1573.8)	2175.8 (1797.8)	0.013
**Δ 17α-Hydroxyprogesterone (pg/mL)**	-10.5 (283.9)	-44.9 (267.3)	-7.3 (336.1)	0.934
**Δ Dihydrotestosterone (pg/mL)**	7.1 (57.5)	0.94 (64.8)	17.1 (45.5)	0.996
**Δ 5α-androstan-3α,17β-diol 3-glucuronide (pg/mL)**	50.8 (262.6)	-5.1 (287.4)	145.7 (171.4)	0.296
**Δ 5β-androstan-3α,17β-diol 3-glucuronide (pg/mL)**	-48.0 (150.7)	-91.2 (156.9)	-36.8 (102.6)	0.358
**Δ 5α-androstan-3α,17β-diol 17-glucuronide (pg/mL)**	-236.8 (409.0)	-374.0 (338.7)	-214.9 (441.6)	0.935
**Δ 5β-androstan-3α,17β-diol 17-glucuronide (pg/mL)**	-800.8 (1602.1)	-730.9 (1700.9)	-866.7 (1049.2)	0.886
**Δ Etiocholanolone Glucuronide (ng/mL)**	-20.1 (14.5)	-20.1 (13.7)	-20.1 (15.7)	0.732
**Δ Androsterone Glucuronide (ng/mL)**	1.6 (8.0)	-0.99 (7.7)	4.9 (9.0)	0.190
**Δ Testosterone Glucuronide (pg/mL)**	-39.2 (81.8)	-43.2 (82.9)	-39.2 (67.9)	0.725
**Δ DHEAS (ng/mL)**	98.0 (291.3)	90.7 (226.2)	98.0 (370.3)	0.779

**Δ Testosterone Sulfate (pg/mL)**	1508.3 (1343.4)	1506.0 (998.2	2093.9 (1702.9)	0.747
**Δ Epitestosterone Sulfate (pg/mL)**	44.2 (140.8)	13.9 (123.0)	83.2 (117.6)	0.575
**Δ Epiandrosterone Sulfate (ng/mL)**	2.1 (18.2)	2.8 (23.4)	2.2 (19.3)	0.488
**Δ Etiocholanolone Sulfate (ng/mL)**	6.5 (40.5)	11.5 (37.5)	1.6 (40.4)	0.308
**Δ Androsterone Sulfate (ng/mL)**	1.7 (58.8)	0.6 (49.7)	9.0 (84.1)	0.572
**Δ T/A4**	-1.9 (3.5)	-0.9 (2.5)	-3.9 (4.2)	0.003

Table S5 – Logistic Regression Analysis Predicting RPE ≥ 4 Based on Pre-Post Steroid Changes. Multivariable logistic regression models were used to assess the association between changes in serum steroid concentrations (Δ) and the likelihood of experiencing a high perceived training load (RPE ≥ 4). Each model was adjusted for relevant training-related covariates, including training type (HL, ML, LL), heart rate exertion, dynamic stress load, and speed intensity. Results are reported as odds ratios (OR), 95% confidence intervals (CI), and p-values. Abbreviations: CI, confidence interval; OR, odds ratio; RPE, rating of perceived exertion; Δ, delta (post-training minus pre-training concentration).

RPE ≥4	OR	95% CI		p-value
Δ E (ng/mL)	0.921	0.751	1.130	0.431
Δ F (ng/mL)	0.956	0.917	0.996	0.032
Δ CORT (pg/mL)	0.998	0.996	1.000	0.060
Δ 11-DF (pg/mL)	0.990	0.982	0.998	0.010
Δ A4 (pg/mL)	0.997	0.992	1.002	0.196
Δ T (pg/mL)	1.002	0.999	1.003	0.074
Δ DHEA (pg/mL)	0.999	0.998	0.999	0.049
Δ 17αOHP (pg/mL)	1.000	0.997	1.004	0.823
Δ DHT (pg/mL)	0.992	0.972	1.013	0.473
Δ 5ααβ-Adiol 3G (pg/mL)	0.999	0.995	1.004	0.721
Δ 5βαβ-Adiol 3G (pg/mL)	0.998	0.994	1.001	0.202
Δ 5ααβ-Adiol 17G (pg/mL)	0.999	0.997	1.002	0.670
Δ 5βαβ-Adiol 17G (pg/mL)	0.999	0.999	1.000	0.298
Δ EtioG (ng/mL)	0.999	0.999	1.000	0.535
Δ AG (ng/mL)	0.999	0.999	1.000	0.060
Δ TG (pg/mL)	1.004	0.990	1.017	0.604
Δ DHEAS (ng/mL)	0.999	0.996	1.004	0.992
Δ TS (pg/mL)	1.000	0.999	1.001	0.479
Δ EpiTS (pg/mL)	1.001	0.995	1.007	0.873
Δ EpiAS (ng/mL)	1.026	0.985	1.068	0.217
Δ EtioS (ng/mL)	1.023	0.999	1.047	0.053
Δ AS (ng/mL)	1.000	0.984	1.016	0.981
